# The Role of Aspirin in the Management of Intracranial Aneurysms: A Systematic Review and Meta-Analyses

**DOI:** 10.3389/fneur.2021.646613

**Published:** 2021-03-30

**Authors:** Shuwen Yang, Tianyu Liu, Yuehui Wu, Nina Xu, Liangtao Xia, Xinyu Yu

**Affiliations:** ^1^Department of Neurosurgery, People's Hospital of Huangpi District, Jianghan University, Wuhan, China; ^2^Department of Neurosurgery, Union Hospital, Tongji Medical College, Huazhong University of Science and Technology, Wuhan, China; ^3^Division of Cardiothoracic and Vascular Surgery, Tongji Hospital, Tongji Medical College, Huazhong University of Science and Technology, Wuhan, China

**Keywords:** aspirin, intracranial aneuryms, aneurysmal subarachnoid hemorrhage, prevention, meta-analysis

## Abstract

**Objective:** To evaluate the association between aspirin use and the risks of unruptured intracranial aneurysm (UIA) growth and aneurysmal subarachnoid hemorrhage (aSAH).

**Methods:** We searched PubMed and Scopus from inception to 1 September 2020. Studies evaluating the associations between aspirin prescription and the risk of UIA growth or the risk of aSAH were included. The study only included patients with intracranial aneurysms. We assessed the quality of included studies using the Newcastle-Ottawa scale. Random-effects meta-analysis was conducted to pool the estimates of effect size quantitatively. Sensitivity analyses using the leave-one-out strategy were performed to identify any potential source of heterogeneity.

**Results:** After a review of 2,226 citations, five cohort studies, two case-control studies, and one nested case-control study involving 8,898 participants were included. Pooled analyses showed that aspirin use, regardless of frequency and duration, was associated with a statistically significantly lower risk of UIA growth (OR 0.25, 95% CI 0.11–0.54; *I*^2^ = 0.0%, *p* = 0.604) and aSAH (OR, 0.37, 95% CI, 0.23–0.58; *I*^2^ = 79.3%, *p* = 0.001) in patients presented with intracranial aneurysms. The results did not significantly change in sensitivity analyses.

**Conclusions:** Summarizing available evidence in the literature, our findings indicate that aspirin use, regardless of frequency and duration, was associated with a statistically significantly lower risk of UIA growth and aSAH in patients with UIA. Well-designed and large-scale clinical trials are needed to help define the role of aspirin as a protective pharmaceutical for UIAs.

## Introduction

According to global statistics, it is estimated that 3% of the adult population has an unruptured intracranial aneurysm (UIA) (1). With the development of non-invasive imaging techniques, an increasing number of UIAs are being detected (2). Despite the further expansion of endovascular techniques and surgical clipping in recent years, the incidence of aneurysmal subarachnoid hemorrhage (aSAH) is relatively unchanged worldwide (3). Small aneurysms (<7 mm) are often left untreated because these patients cannot benefit from existing treatments, and the risk of aneurysm rupture does not outweigh the risk of morbidity and mortality from treatment complications for these aneurysms. Due to the non-negligible rate of aneurysm growth, regular follow-up with imaging surveillance to assess change in size and morphology is indicated (4–6). However, the continuous growth of an intracranial aneurysm results in subarachnoid hemorrhage (SAH), which has a mortality of 35%, and leads to serval serious complications (7). Thus, there is an urgent need for a non-invasive pharmaceutical treatment that can mitigate the risk of UIA growth.

Recently, accumulative evidence has suggested that inflammation plays a critical role in the structural deterioration of the IA wall and its subsequent rupture (8). Several observational studies have linked a representative non-steroidal anti-inflammatory drug-aspirin use with a slower rate of IA growth and lower risk of aSAH (4, 9–15). Aspirin has been widely prescribed as a standard secondary preventative agent in patients with risks of cardio- and cerebrovascular diseases. If aspirin is proved to have a beneficial effect on the risk of UIA growth with an acceptable safety profile, it could be a promising treatment option for this indication. As such, we conducted this systematic review and meta-analysis including patients with intracranial aneurysms to evaluate the association between aspirin use and risk of UIA growth and aSAH.

## Methods

### Search Strategy

We conducted this systematic review and meta-analysis following the Preferred Reporting Items for Systematic Review and Meta-Analysis guidelines 2009 (16). This systematic review and meta-analysis was not registered in the PROSPERO database. We thoroughly searched PubMed and Scopus from inception to 1 September 2020. A combination of search terms related to aspirin use (i.e., acetylsalicylic acid,) and outcomes of interest (i.e., occurrence of aSAH, growth of UIA) were used in the search strategy. We also searched the references of the included articles for further information. The details of the search strategy for each of the databases are included in Supplementary Materials.

### Inclusion Criteria

Two collaborators (SY. and LX) individually screened the studies from two databases for eligibility according to predefined selection criteria: (i) the research design was cohort, case-control, or cross-sectional study; (ii) the study population was patients with UIAs and aspirin was the exposure factor; (iii) the primary outcome contained the prevalence of UIA growth or aSAH; and (iv) the study reported the odds ratio (OR) and corresponding 95% confidence intervals (CIs) (or OR and 95%CI can be manually derived from the study). Reviews, animal studies, clinical trials, case reports, commentaries were excluded. Disagreements were solved in a discussion with a senior author (XY.).

### Data Extraction

Two investigators attentively screened the titles and abstracts of articles and excluded irrelevant studies after duplicates were removed. After the first-round review, the same investigators retrieved full reports of those potentially eligible studies for details independently and then included studies that met the inclusion criteria. The disagreement was resolved in discussions with a third reviewer.

Data were extracted from retrieved articles by two reviewers independently. Details on the name of the first author, year of publication, region, study design, age and gender ratio of participants, exposures, primary outcomes, controls, OR with 95% Cis, and covariates adjusted rates, if available, were recorded.

### Quality Appraisal

We appraised included studies using the Newcastle-Ottawa Scale 10, which is a nine-point scoring system used to assess the quality of non-randomized studies included in a systematic review/meta-analysis. A high-quality study was defined as a study with at least seven points. All items were independently assessed by two investigators with disagreements resolved by group discussion.

### Statistical Analysis

We preferred to pool adjusted ORs from the primary studies; otherwise, we used the unadjusted estimates. A random-effects model was used to pool the effect estimates and *I*^2^ statistic was used to evaluate heterogeneity (0–100%). We considered *I*^2^ < 50% as low heterogeneity, *I*^2^ of 50–75% as moderate heterogeneity, and *I*^2^ > 75% as statistically high heterogeneity. We performed sensitivity analyses using a “leave-one-out” strategy to clarify the potential sources of the heterogeneity between included studies which may result from differences in the study population, intervention, or comparators. Also, we planned to assess for publication bias by the Egger test and funnel plots. All analyses were conducted in Stata version 11.

## Results

### Literature Search

**Figure 1:**
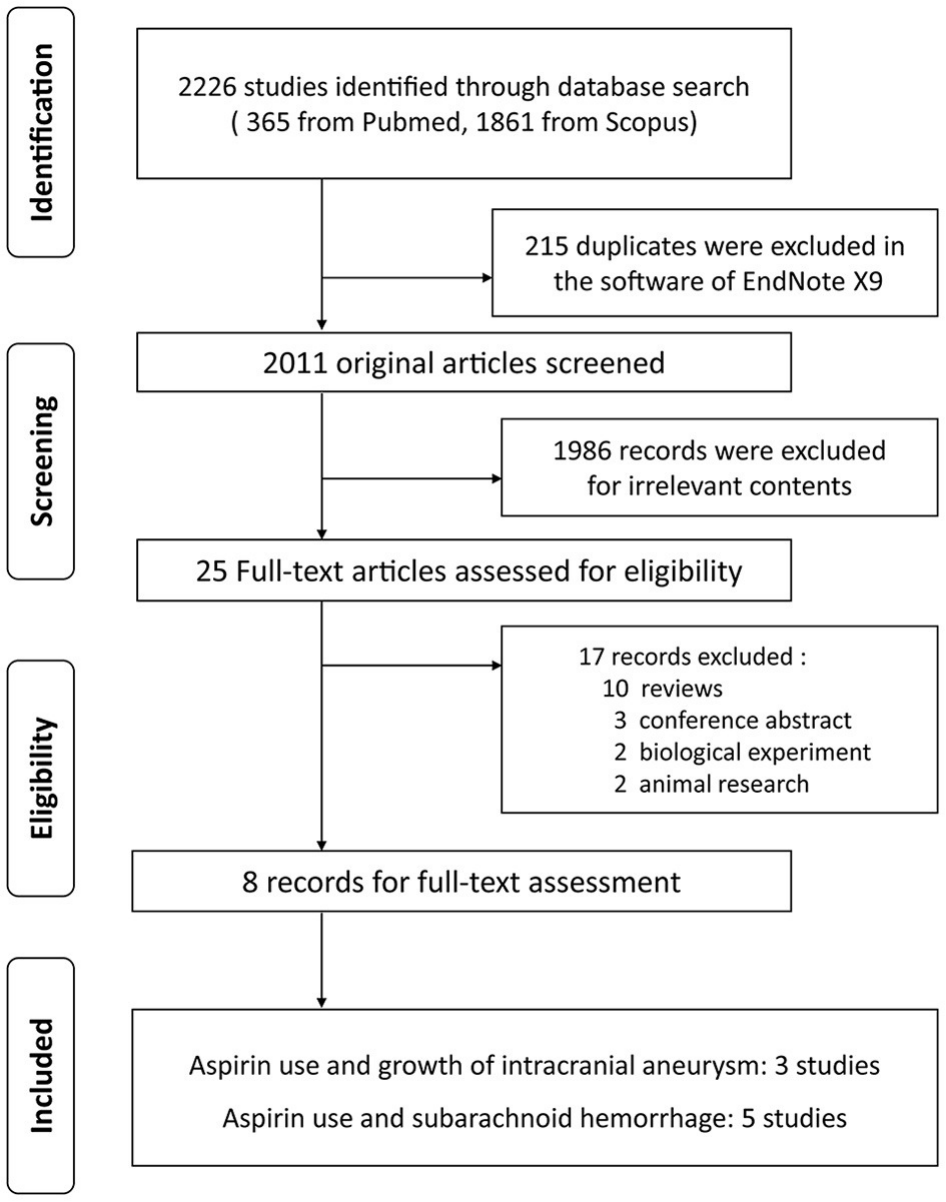
Study screening flowchart.

Figure 1 displays the flow chart of our study. We identified 2,226 citations from PubMed and Scopus. Eight studies met the inclusion criteria and provided data with 8,898 distinct participants: one prospective cohort study reported associations between aspirin use and UIA growth/rupture; four retrospective studies of either a prospectively maintained database, a patient cohort, or a consecutive series, indicated a negative relationship between aspirin use and UIA growth or aSAH; two case-control studies and 1 nested case-control study discussed the relationship between aspirin use and risk of aSAH. Table 1 illustrates the detailed characteristics of the included studies, whose quality was carefully assessed by the Newcastle-Ottawa Scale (see Table 2).

**Table 1:** Characteristics of included studies in the systematic review.

**Study Authors and Published Year (location)**	**Study design**	**Inclusion criteria for participants**	**Definition of aspirin users**	**Number of Cases in the exposure group**	**Follow-up Duration, mean**	**Definition of outcomes**	**Adjusted estimate, (95% CI)/other outcomes**	**Adjustment of covariates**
Weng et al. (17)	Prospective cohort study	Patients with UIAs <7 mm and concurrent ischemic cerebrovascular diseases between Jan 2016 and Dec 2019. (*n* = 272)	**Aspirin users** were defined as those who reported aspirin use at least 3 × per week, including standard- and low-dose aspirin. **Non-aspirin users** were those who used no aspirin.	113	19.6 months	**The primary outcome**: Aneurysm growth, which was defined as [1] growth ≥1.0 mm in at least 1 direction by identical imaging modalities, [2] growth ≥0.5 mm in 2 directions by identical imaging modalities, and [3] an indisputable change in aneurysm shape. **The secondary outcome:** UIA rupture. The diagnosis of aneurysm rupture was confirmed by preoperative CT, MR imaging, cerebrospinal fluid analysis, or a neurosurgeon during operation.	**The primary outcome**: **HR, 0.29 (0.11–0.77)** The cumulative annual growth rates were as high as 40.0 and 53.3 per 100 person-years in the high-risk patients (>1 risk factor) with and without aspirin, respectively. **The secondary outcome**: No aneurysm rupture	Age, female sex, hyperlipidemia, pretransient ischemic attack, or ischemic stroke
Zanaty et al. (9) (Japan)	A retrospective review of a prospectively maintained database	[1] Patients harbored multiple saccular IAs; [2] At least one primary aneurysm was treated with coiling, stent-assisted coiling, flow diversion, or microsurgical clipping;[3] The remaining aneurysms were ≤ 5 mm in size and observed for growth; and [4] At least 5 years of follow-up from the initial treatment was available. (*n* = 146)	**Aspirin users** were defined as those who reported aspirin use ≥81 mg daily. **Non-aspirin users** were those who used no aspirin.	69	More than 5 years	**The primary outcome**: the interval growth of any remaining untreated aneurysms that later required treatment. Growth was defined as an increase in the size of the aneurysm ≥1 mm. All aneurysms that demonstrated growth underwent treatment regardless of size.	**The primary outcome**: **OR, 0.19 (0.05–0.63)**	Patient sex and age, aneurysm size and location, rupture status of the designated primary aneurysm at the initial encounter, hypertension, diabetes mellitus, hypercholesterolemia, use of other anticoagulant or antiplatelet medication, family history of IAs, drug abuse, polycystic kidney disease.
Serrone et al. (14) (United States)	A retrospective review of a patient cohort	Patients are seen in the clinic with the diagnosis of an untreated UIA and at least 1 follow-up clinic visit or consultation. (*n* = 192)	**Aspirin users** were defined as those who reported aspirin use. **Non-aspirin users** were those who used no aspirin.	120	11.5	**The primary outcome:** Aneurysm growth or de novo aneurysm formation	**The primary outcome**: **OR, 0.72 (0.29–1.81)**	NA
Gross et al. (11) (United States)	A retrospective review of a consecutive series	Patients with at least one cerebral aneurysm seen by the neurosurgical service during the study period. (*n* = 717)	**Aspirin users** were defined as those who reported aspirin use (81 or 325 mg). **Non-aspirin users** were those who used no aspirin.	32	7 years	**The primary outcome:** aneurysmal subarachnoid hemorrhage	**The primary outcome**: **OR, 0.58 (0.38–0.90)**	NA
Can et al. (12) (United States)	Case-control study	Patients who were diagnosed with an intracranial aneurysm between 1990 and 2016 (*n* = 4,619).	**Aspirin users** were defined as those who reported aspirin use. **Non-aspirin users** were those who used no aspirin.	99	NA	**The primary outcome:** aneurysmal subarachnoid hemorrhage	**The primary outcome**: **OR, 0.60 (0.45–0.80)**	Age, sex, and race, and comorbid conditions, including hypertension, coronary artery disease, myocardial infarction, and atrial fibrillation, antihypertensive medication use, family history of aneurysms or SAH, and current tobacco and alcohol use.
Hostettler et al. (13) (United Kingdom)	Case-control study	Patients with aneurysmal SAH or unruptured aneurysm without previous SAH enrolled in the Genetic and Observational Subarachnoid Hemorrhage study (*n* = 2,334).	Aspirin use was defined by patient self-reporting or available documentation on regular intake at the time of either admission with aneurysmal SAH or of being diagnosed with an unruptured aneurysm	120	NA	**The primary outcome:** aneurysmal subarachnoid hemorrhage	**The primary outcome**: **OR, 0.28 (0.20–0.40)**	Age, sex, ethnicity, smoking status, use of antihypertensive medication, hypercholesterolemia, aneurysm location, aneurysm size.
Nisson et al. (15) (United States)	Retrospective cohort study	Patients who underwent surgery for intracranial aneurysm between January 2010 and April 2013 at a tertiary academic medical center (*n* = 347).	**Aspirin users** were defined as those who reported aspirin use. **Non-aspirin users** were those who used no aspirin.	9	11.5	**The primary outcome:** aneurysmal subarachnoid hemorrhage	**The primary outcome**: **OR, 0.18 (0.09–0.39)**	NA
Hasan et al. (10) (United States)	Nested case-control study	[1] Patients must have at least one UIA, which may or may not be symptomatic. [2] Patients who have had a ruptured aneurysm at another location that was isolated, trapped, clipped, or treated through endovascular obliteration must be able to care for themselves after the aneurysmal treatment according to a follow-up evaluation at 30 days of post-treatment. (*n* = 271)	**Aspirin users** were defined as those who reported aspirin use based on questionnaires. **Non-aspirin users** were those who used no aspirin.	19	5 years	**The primary outcome:** UIA rupture. The adjudicated hemorrhage events were defined as a primary hemorrhage if either: [1] a definite or highly probable SAH of aneurysmal or unknown etiology or [2] a definite or highly probable intracranial hemorrhage determined to be of aneurysmal etiology.	**The primary outcome**: **OR, 0.27 (0.11–0.67)**	Age, sex, UIA enrollment group, participating center location, multiple aneurysm, hypertension, cardiac valvar disease, atrial fibrillation-flutter, other cardiac arrhythmias, congestive heart failure, myocardial infarction, family history of intracranial aneurysm hemorrhage, smoking, alcohol consumption, use of anticoagulants, history of aneurysms, interaction smoking and hypertension.

*OR, odds ratio; CI, confidence interval; NA, not available*.

**Table 2:** Newcastle-Ottawa scale for assessing the quality of included studies.

**Study design**	**Author, year (Pubmed ID)**	**Selection (Max=4)**	**Comparability (Max=2)**	**Exposure (Max=3)**	**Overall quality score (Max=9)**
Case-control study	Can et al. (12) (30135253)	4	2	2	8
	Hostettler et al. (13) (28973585)	4	2	2	8
	Hasan et al. (18) (21980208)	4	2	2	8
**Study design**	**Author, year (Pubmed ID)**	**Selection (Max=4)**	**Comparability (Max=2)**	**Outcome (Max=3)**	**Overall quality score (Max=9)**
Cohort study	Weng et al. (32878566)	4	2	3	9
	Nisson et al. (15) (31857268)	4	1	3	8
	Zanaty et al. (9) (31662579)	4	2	3	9
	Serrone et al. (14) (26967775)	4	1	2	7
	Gross et al. (11) (23548847)	4	1	3	8

### Outcome Measure

#### Aspirin Use and the Risk of UIA Growth

**Figure 2:**
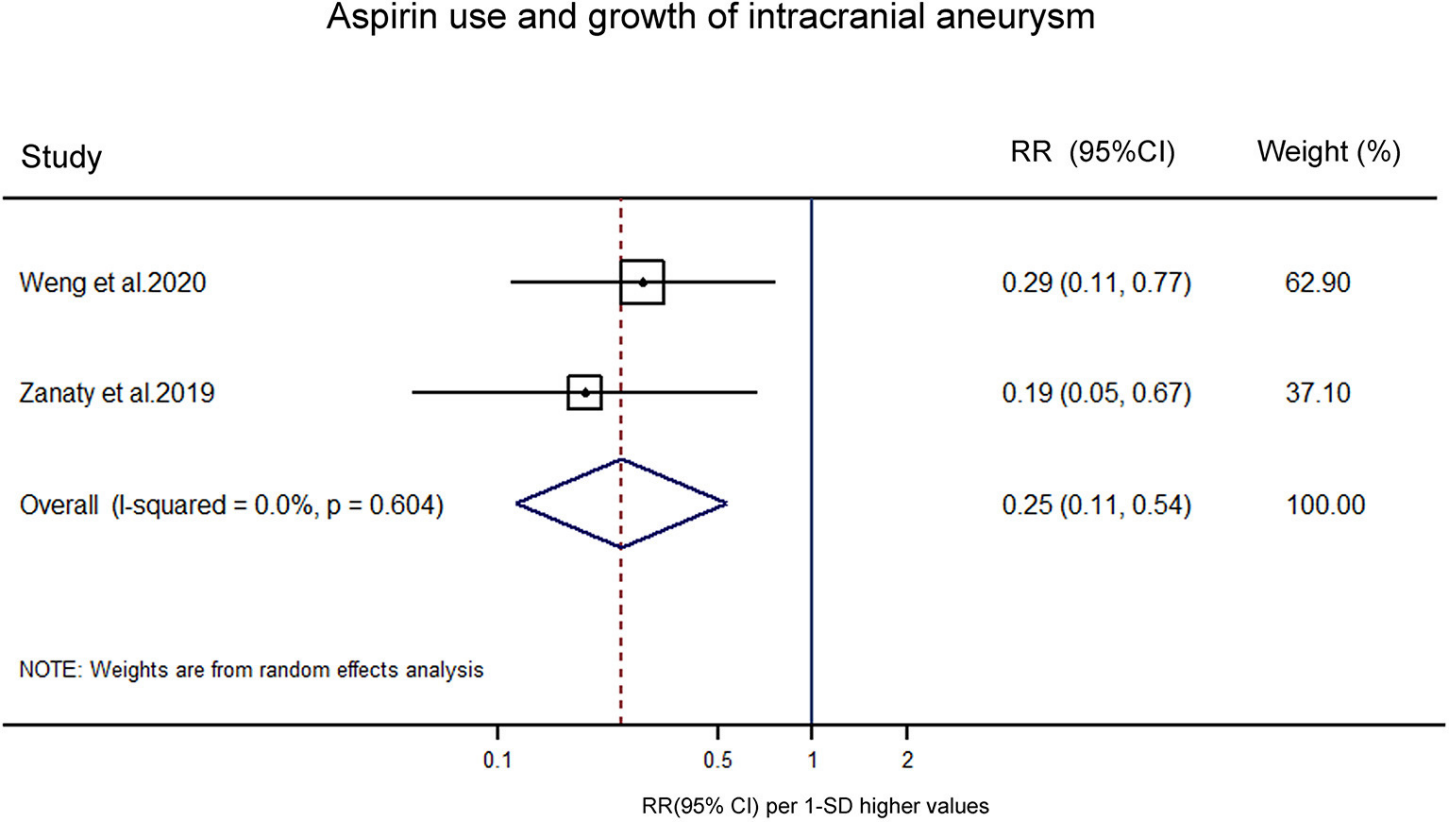
Forest plot for an association between aspirin use and growth of intracranial aneurysm. OR, odds ratio; CI, confidence interval.

Three studies reported associations between aspirin use and UIA growth. Although Serrone et al. identified a relatively lower risk in aspirin users (OR 0.72, 95% CI 0.29–1.81), their primary outcome was UIA growth or *de novo* aneurysm formation (14). Thus, we excluded it from the pooled analyses. Combining findings from the other two studies suggested that aspirin use, regardless of frequency and duration, was associated with a significantly lower risk of UIA growth (OR 0.25, 95% CI 0.11–0.54) (Figure 2). No significant heterogeneity was observed (*p* = 0.604).

#### Aspirin Use and the Risk of aSAH in Patients With UIA

**Figure 3:**
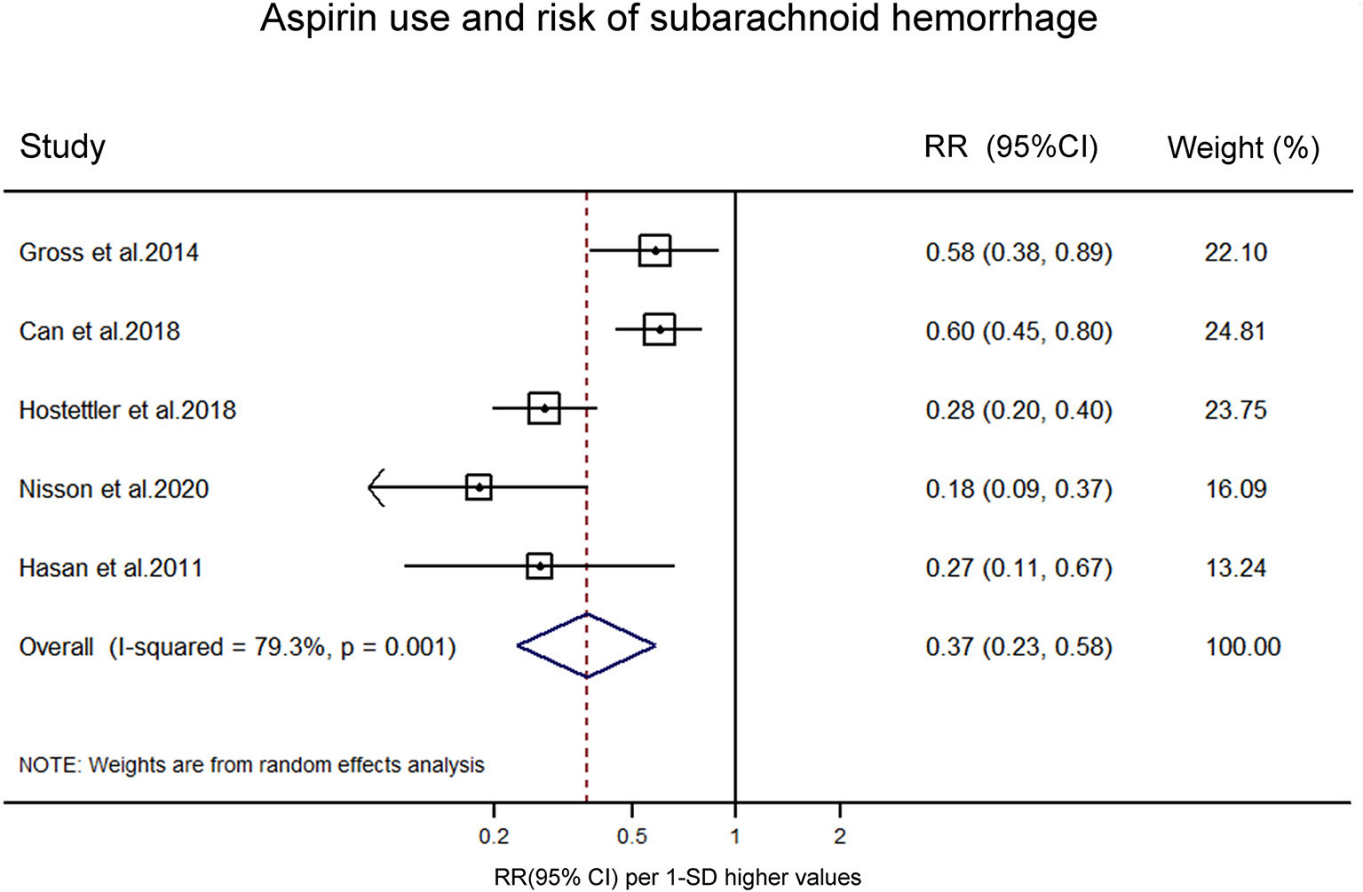
Forest plot for an association between aspirin use and risk of subrachnoid hemorrhage. OR, odds ratio; CI, confidence interval.

**Figure 4:**
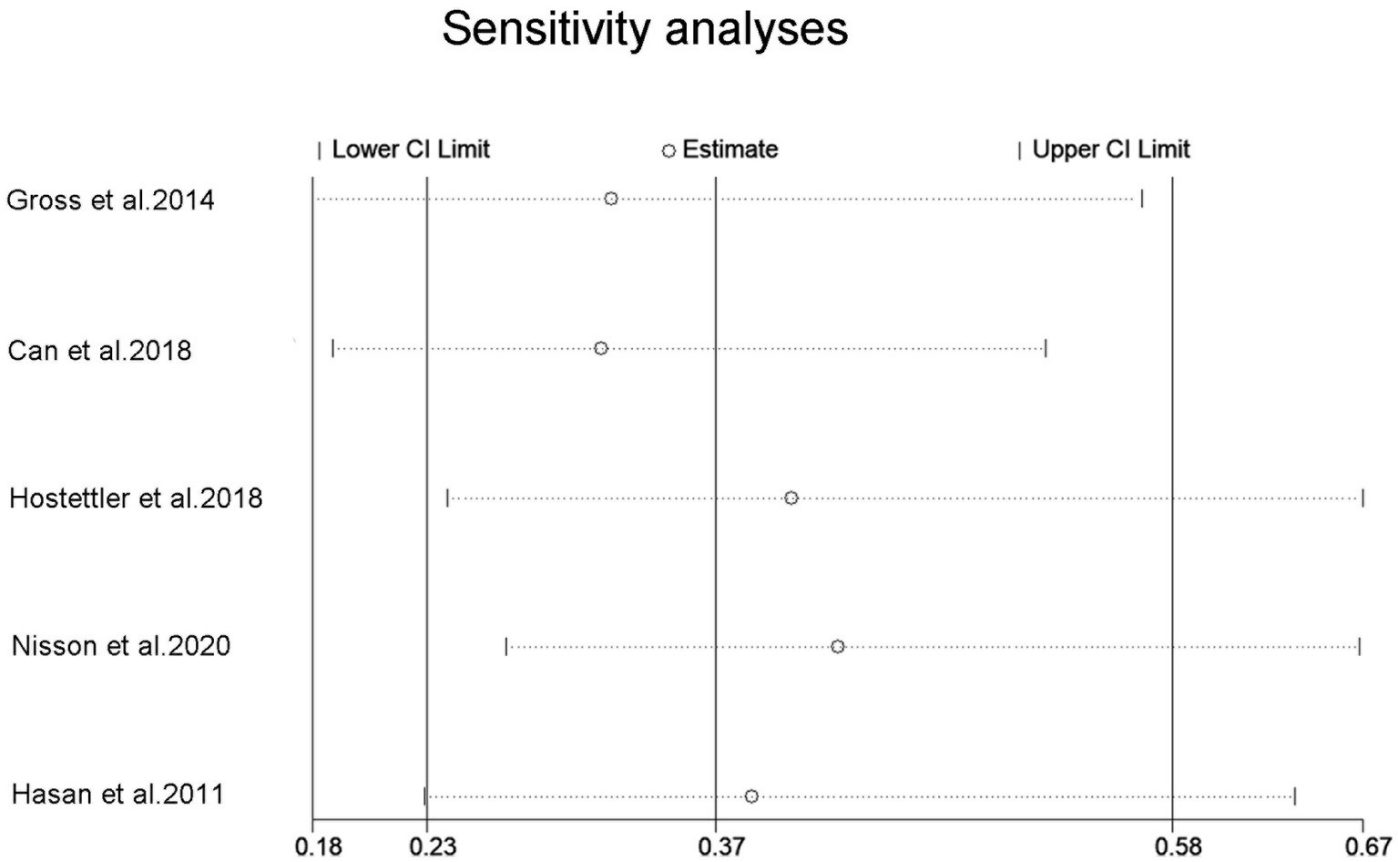
Sensitivity analysis for an association between aspirin use and risk of subrachnoid hemorrhage. OR, odds ratio; CI, confidence interval.

Five studies reported on the association between aspirin use and risk of aSAH in patients with UIA. A meta-analysis was conducted to pool estimates of aspirin use and the risk of aSAH in UIA patients, resulting in an OR of 0.37 (95% CI, 0.23–0.58) (Figure 3). Significant heterogeneity was tested out in the included studies (*p* = 0.001). We then conducted a sensitivity analysis using a leave-one-out strategy. Figure 4 showed the corresponding pooled ORs when one study was excluded from the final analysis. The results remained stable when any specific study was excluded from the pooled analysis, indicating that aspirin use was associated with a lower risk of aSAH in patients with UIA despite the high heterogeneity in studies.

## Discussion

In the present systematic review and meta-analysis, we summarized all available epidemiological evidence, using data from 8,898 participants involving 581 cases in the aspirin users to help clarify the association between aspirin use and UIA growth or aSAH in UIA patients. Our results showed that aspirin use, regardless of frequency and duration, was associated with a statistically significant decreased risk of UIA growth (OR 0.25, 95% CI 0.11–0.54; *I*^2^ = 0.0%, *p* = 0.604) and a significantly lower risk of aSAH (OR, 0.37, 95% CI, 0.23–0.58; *I*^2^ = 79.3%, *p* = 0.001) in patients with UIAs. The results of this study suggest that aspirin could play a role in reducing the risk of intracranial aneurysm expansion and the risk of aSAH, and aspirin could be a potential drug to treat intracranial aneurysms.

Two previous meta-analyses have discussed the effect of aspirin prescription on the risk of aSAH (6, 19). Both meta-analyses found no significant difference between aspirin users and non-aspirin users regarding the risk of aSAH (OR, 1.00; 95% CI, 0.81–1.24, *p* = 0.99, and OR, 0.981,95% CI, 0.773–1.312, *p* = 0.897). However, neither of the two meta-analyses focused on the risk of aSAH in the specific patient group with intracranial aneurysms, which may attenuate the possible protective effect of aspirin on IA rupture and aSAH in UIA patients. Moreover, better concomitant risk factor management in the UIA patients, particularly blood pressure control, might contribute to the lower risk of UIA progression. Besides, Phan et al. reported a significant association between short-term use of aspirin (<3 months) and risk of aSAH (OR, 1.61; 95% CI, 1.20–2.18, *p* = 0.002) (6). Qian et al. also reported that short-term use of aspirin (<3 months) was associated with an elevated risk of aSAH (OR, 1.697, 95% CI, 1.175–2.452, *p* = 0.005) (19). They concluded that when prescribing aspirin for prophylactic use, particularly with known UIAs, its inherent bleeding risks should be taken into consideration, especially in the short term. Several population-based studies have explored the association between antiplatelet therapy and SAH, reaching conflicting results (20, 21). Recently, Weng et al. provided Class III evidence in a prospective, multicenter cohort that for patients harboring UIAs <7 mm with ischemic cerebrovascular disease, aspirin does not increase the risk of aneurysm rupture (17). Together with our findings, we believe that the benefit of aspirin uses in this specific population outweighs the possibly increased risk of aSAH.

Both animal experiments and human clinical studies indicate that vascular remodeling and inflammatory cascades are crucial in the formation, progression, and rupture of IAs (22). Abnormal wall shear stress-activated the PGE2 (prostaglandin E2) -EP2 (prostaglandin E receptor 2) pathway in endothelial cells (ECs) at the early stage of cerebral aneurysm formation (23, 24). Subsequently, vascular smooth muscle cell apoptosis and migration, accompanied by inflammatory cell infiltration, resulted in degradation of the vascular wall, leading to the progression, and eventual rupture of IAs (22). Hasan et al. found in a small patient group that cyclooxygenase-2 (COX-2) and microsomal prostaglandin E2 synthase-1 (mPGES-1) are expressed in human cerebral aneurysms and expression increases in ruptured aneurysms (25). Thus, drugs targeting molecules involved in the above process might have potential therapeutic effects. As a commonly used preventative agent in patients with risks of cardio- and cerebrovascular diseases, aspirin has been shown to have inhibitory effects on several inflammatory mediators such as COX-2 and mPGES-1, making it one of the promising drugs for decreasing UIA growth and rupture (10). Several groups have proved that acetylsalicylic acid (ASA) was associated with a slower IA growth rate and lower IA rupture or aSAH rate in mice IA-induction models, suggesting the protective effect of ASA against IA rupture (8). Moreover, Hasan et al. demonstrated a decreased expression of inflammatory cells and markers such as COX-2 in a small randomized sample of patients with unruptured aneurysms who underwent microsurgical clipping after 3 months of aspirin treatment (18). More researches should be conducted to further elucidate the underlying mechanisms of this issue.

The present study was constrained by several limitations. Firstly, the number of included studies was relatively low, especially for the meta-analysis on UIA growth. Secondly, all eligible data included in the meta-analysis were extracted from observational studies and most studies were retrospective. Last but not least, heterogeneity among studies suggests that the effect of aspirin on UIA growth and rupture should be further confirmed by clinical trials. Re-analyzing existing non-randomized data using advanced statistical techniques (i.e., inverse probably of treatment weighting) could better explore this association as well.

## Conclusion

Summarizing available evidence in the literature, our findings indicate that aspirin use, regardless of frequency and duration, was associated with a statistically significant decreased risk of UIA growth and aSAH in UIA patients. Aspirin might be a potential drug for the treatment of intracranial aneurysms. Well-designed, large-scale clinical trials are needed to help definitively define aspirin's role as a protective pharmaceutical for UIAs.

## Author Contributions

SY, LX, and XY contributed to the conception or design of the work and contributed to the acquisition, analysis, or interpretation of data for the work. SY and LX drafted the manuscript. TL, YW, and NX critically revised the manuscript. All gave final approval and agree to be accountable for all aspects of work ensuring integrity and accuracy.

## Conflict of Interest

The authors declare that the research was conducted in the absence of any commercial or financial relationships that could be construed as a potential conflict of interest.
